# Simple Synthesis of 17-β-*O*-hemisuccinate of Stanozolol for Immunoanalytical Methods

**DOI:** 10.3390/molecules25092019

**Published:** 2020-04-26

**Authors:** Silvana Casati, Roberta Ottria, Pierangela Ciuffreda

**Affiliations:** Dipartimento di Scienze Biomediche e Cliniche “Luigi Sacco”, Università degli Studi di Milano, Via G.B. Grassi 74, 20157 Milano, Italy; silvana.casati@unimi.it (S.C.); roberta.ottria@unimi.it (R.O.)

**Keywords:** steroids, stanozolol, metabolism, synthesis, anabolic–androgenic steroid

## Abstract

The use of doping in sports is a global problem that affects athletes around the world. Among the different methods developed to detect doping agents in biological samples, there are antibody-based methods that need an appropriate hapten design. Steroids with a hydroxyl group can be converted to the corresponding hemisuccinates. A novel approach to the synthesis of 17β-*O*-hemisuccinate of the common doping agent stanozolol is described here. Acylation of stanozolol with methyl 4-chloro-4-oxobutyrate/4-dimethylaminopyridine, followed by mild alkaline hydrolysis with methanolic sodium hydroxide at room temperature, gave the simultaneous protection and deprotection of pyrazole-nitrogen atoms. The proposed new synthetic method allows the desired hemisuccinate derivative to be obtained in only two steps, and with a good total yield starting from stanozolol.

## 1. Introduction

Doping in competitive sports is a threat not only for ethics in sports but also for the athlete’s health. The World Anti-Doping Agency (WADA) was founded to counteract this despicable practice, but there are some critical issues [1]. The problem of anti-doping tests has a changing legal face, as it touches forensic, health, and even agricultural regulations [2]. Cheap and ready-to-use methods are necessary for efficient control of anabolic–androgenic steroids (AAS) and similar compounds. The ideal method for screening of anabolic steroids should be fast, simple, cost-effective, easy to perform, and should enable measurement in a small volume of biological fluid [3]. Food safety and accredited WADA laboratories use HPLC-MS and GC-MS as the most common analytical methods to routinely control the use of these substances [4,5,6,7,8]. In addition, the literature presents a few other analytical methods that couple HPLC or GC techniques with immunochemical detection [9,10] and a chemiluminescent immunoassay method [11]. The analysis of anabolic steroids presents some difficulties related to their neutral and relatively nonpolar nature, together with the very low levels that must be detected and the high levels of naturally occurring steroids usually present in the samples. Antibody-based detection methods offer rapid, simple, and cost-effective alternatives for analytical measurements. They are one of the more sensitive assays available because of the high affinity of antibodies and fulfill most of the criteria suitable for routine high-throughput screening. In addition, an appropriate hapten design allows raising antibodies, obtained after immunization of selected animals, with tailored features and cross-reactivity patterns that make possible registering both the parent molecules and their metabolites simultaneously. For the purpose of immunization, an immunogen, a carrier protein with bound molecules of target steroid via a defined spacer, is used.

Steroids conjugated to macromolecules can elicit anti-steroid antibodies and serve as solid-phase antigens for the immunological detection of the hormones [12]. Different reactions are used to link these haptens to the macromolecular carrier, depending on the functional groups of the molecules. Condensation between carboxylic and amino groups is the most widely employed procedure, applicable also to compounds that do not possess these functional groups originally [13]. More specifically, steroids with a hydroxyl group can be converted to their corresponding hemisuccinates, with the hemisuccinate linker that represents one of the typically used spacers in this respect [14].

In the literature, there are several direct and indirect methods for the preparation of hemisuccinates. The usual synthesis of steroidal hemisuccinates by the direct method treats the corresponding alcohols with succinic anhydride in pyridine [15], at room or elevated temperature. This method, applied for example to testosterone, is focussed on the base catalysis of pyridine but cannot be applied to sensitive and reactive substrates [16]. Another direct method, not examined on steroids, employs anhydride and, as catalyst, bismuth triflate (Bi(OTf)_3_). The Bi(OTf)_3_/acid anhydride protocol was so powerful that sterically hindered or tertiary alcohols could be acylated [17]. Finally, the ability of hydrolases to accomplish one-step syntheses of specific steroid hemisuccinates was also investigated. In this respect, 3,17-β-estradiol was regioselectively acylated with succinic anhydride at the 17β-hydroxyl by the action of subtilisin Carlsberg in organic solvent [18].

Indirect methods for the synthesis of hemisuccinates of steroids bearing a tertiary alcohol group, use methylmagnesium bromide to form a steroidal alkoxide and, methyl succinyl chloride as an acylating reagent. The corresponding hemisuccinate is then obtained by the hydrolysis of monomethyl ester [19]. Another indirect method, described only for hemisuccinate preparation of steroids bearing a secondary alcohol group, consists of the reaction of the steroidal alcohol with 4-(2,2,2-trichloroethoxy)-4-oxobutanoic acid in benzene in the presence of N,N′-dicyclohexylcarbodiimide (DCC) and 4-dimethylaminopyridine (DMAP) that affords the blocked ester formation, and the following release of the hemisuccinate at 0 °C with zinc powder in a tetrahydrofuran-acetic acid-water mixture [14].

**Scheme 1:**
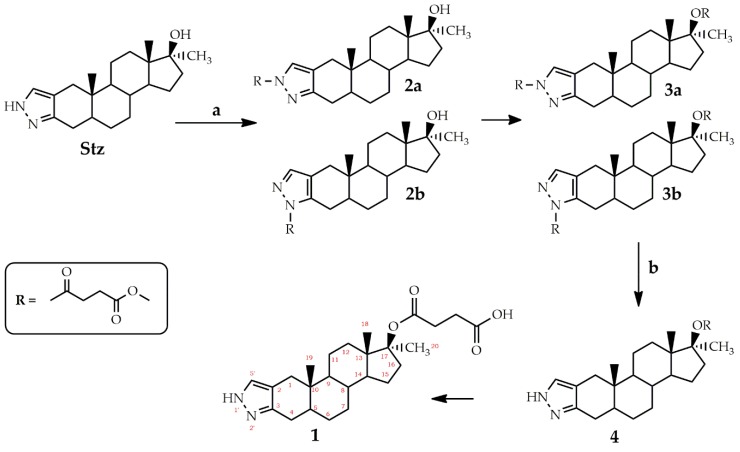
Synthesis of **Stz** hemisuccinate (**1**): (a) methyl 4-chloro-4-oxobutyrate, DMAP, CH_2_Cl_2_; (b) NaOH, MeOH.

Stanozolol (**Stz**; Scheme 1), first developed in 1959 [20], is one of the most important synthetic anabolic steroids used to increase human athletic performance, and as a growth promoter in cattle and horses for racing [21]. Several reports document its abuse by athletes to help build muscle mass, boost acceleration, and recover faster from workouts [22]. Beyond illegal uses, we should also recall that **Stz** has substantial fibrinolytic properties and has been effective in the treatment of serious disorders like the Raynaud phenomenon and cryptofibrinogenemia [23]. Other therapeutic uses of **Stz** include treatment of AIDS wasting syndrome [24], aplastic anemia, and hereditary angioedema [25]. It has also been indicated as an adjunct therapy for the treatment of vascular disorders and growth failures, which arise as a result of other medical conditions. Otherwise, the illicit use of AAS in nutritional supplements is a cause of concern [26], so it is crucial to monitor this group of substances. The common analytical methods for determination and quantification of AAS, their metabolites, and **Stz** and its major metabolites such as 3′-hydroxy-stanozolol, 3′-hydroxy-17-epistanozolol, 4β-hydroxy-stanozolol, or 16-hydroxystanozolol, in both powders or liquid matrices as urine samples, include GC-MS and LC-MS-MS based approaches [21,27]. On the other hand, like most other AAS, **Stz** has poor gas chromatographic behavior. Moreover, these methods require authentic standards, expensive instrumentation, and sample analysis needs expert operators and adequately equipped laboratories. In addition, low urinary excretion and renal clearance render these molecules difficult to be detected in urine samples leading to the need for very high sensitive analytical methods. This is due to the rapid metabolization, leading to low concentration levels of the parent compounds found in urine. Instead, ELISA (enzyme-linked immunosorbent assay) is a commonly used analytical assay, as it enables parallel analysis of multiple samples in a short time, utilizing a simple procedure, and appears to be a useful cost-effective screening tool for the preselection of suspect samples for further instrumental approaches, which are, however, irreplaceable in identification and quantification of particular steroids [28]. As previously reported, a carrier protein with bound molecules of the target steroid via a defined spacer is used to elicit anti-histone antibodies [29]. This is also the case for **Stz**, where a specific derivative, with an appropriate spacer, is necessary to build up an efficient antibody for the preparation of an ELISA kit for **Stz** and its metabolites analysis.

Herein, we report on a new synthesis of 17β-*O*-hemisuccinate of **Stz** in only a two-step reaction with 55% overall yields. The simple use of methyl 4-chloro-4-oxobutyrate as the acylating agent to obtain hemisuccinates of steroid alcohols is, to our knowledge, unprecedented in the literature.

## 2. Results and Discussion

The analysis of anabolic steroids is of particular interest for their use both in farms for animal growth promotion and in sports, as AAS have been prohibited substances since 1976. Unfortunately, the analysis of AAS presents some complexities due to their chemical nature, together with the low levels in bio matrices due to the metabolism and the concomitant high level of natural steroids that often act as interfering agents. Moreover, to achieve efficient control of AAS, the knowledge of the metabolic pathway is mandatory, allowing us also to recognize metabolites. In particular, for **Stz,** the 16β-hydroxy-stanozolol (**16-OH Stz**) has been identified as the major urine metabolite in cattle [30] and humans [31,32]. In addition, **16-OH Stz** is principally secreted as glucuronide or glucuronide of the sulfate conjugate, as often happens for **Stz** and other steroids. All these considerations highlight the importance of designing and producing the appropriate hapten to obtain, for analytical applications, antibodies able to recognize both parent molecules and their metabolites.

**Figure 1:**
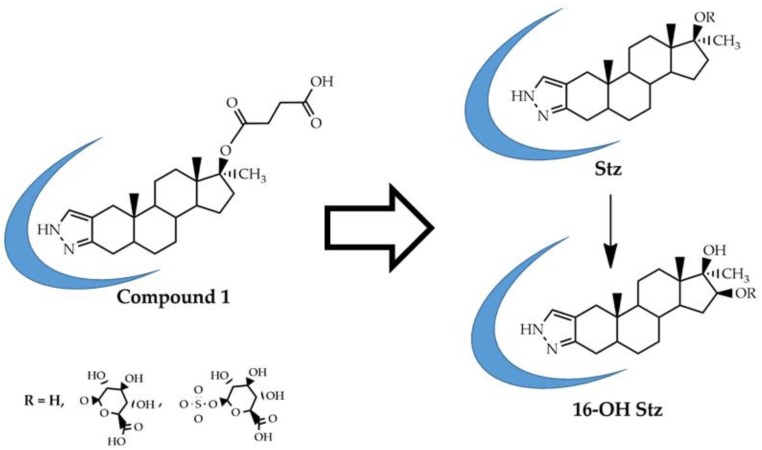
Chemical structures of compound **1**, **Stz**, **16-OH Stz**, and their glucuronides derivatives. In light blue are indicated the expected recognition positions maintained in these chemical structures.

Therefore compound **1**, with a spacer arm at position 17β-OH, was designed to expose the pyrazole ring to the immune system. The selection of the pyrazole moiety, common in the **Stz** and **16-OH Stz** structures, as recognition region site will allow detection both **Stz** and its major urinary metabolite **16-OH Stz,** not only in their natural form but also as glucuronide derivatives or glucuronide of the sulfate conjugates (Figure 1).

With this premise, we addressed the preparation of compound **1** from **Stz** and exploited the 17β-hydroxy group to introduce the linker hemisuccinate.

As **Stz** has more than one reactive group is present on the molecule, the low regioselectivity of this transformation makes it necessary to introduce additional masking and unmasking steps.

There are two reported examples for the synthesis of 17-*O*-hemisuccinate of **Stz**. The first one [33] passes through the synthesis of the oxymetholone hemisuccinate, achieved in two steps by the formation of the carbomethoxy butyrate at the 17-OH of the oxymetholone and the following hydrolysis of the carbomethoxy moiety in mild alkaline conditions. Then, compound **1** is obtained from 3-ketosteroid by the introduction of a 2-formyl group at C-3, followed by condensation of the resulting 3-keto-aldehyde moiety of oxymetholone hemisuccinate with hydrazine hydrate. This first synthesis is a time-consuming procedure that requires four reaction steps, each followed by several crystallizations to isolate the pure intermediate, leading to poor final yields. In the second method [34], the pyrazole-nitrogen atoms have to be protected as Boc derivative in order to avoid its nucleophilic attack in the following step, i.e., the acylation of the 17β-hydroxy group. Then the 2′-Boc derivative is separated and subjected to reaction with 2-(trimethylsilyl)ethyl hydrogen butanedioate and ethyl-3-(3-dimethyl-aminopropyl)-1-carbodiimide hydrochloride (EDC) coupling reagent to give the **Stz** hemisuccinate. Finally, a microwave-assisted reaction was performed in tetrahydrofuran at high temperature and with an excess of TBAF to remove in one-step both 2′-Boc and trimethylsilyl protective groups. Following this second approach, it is worth mentioning that the protection with Boc, that gave two regioisomers in similar yields, and the next selection of the 2′-Boc isomer, leads unavoidably to the halving of the quantity of the final product.

In order to obtain an easier and cheaper synthetic procedure, able to give good yields, we investigated the feasibility of reducing the number of reaction steps using succinate moiety as both protecting group, and spacer linker added respectively at the pyrazole-nitrogen atom and a hydroxyl group in position 17β of **Stz**.

It is known that pyrazole-nitrogen atoms are readily acylated, and it has been demonstrated that the N-acyl groups in *O*,*N*-diacyl steroid-[3,2-c] pyrazoles are sufficiently labile to be removed quite easily and selectively at room temperature [35]. Moreover, despite the inert character of tertiary alcohol in the 17 position, it is possibly the formation of the ester bond [12,13,14].

Herein, we report for the first time the transformation of **Stz** to desired stanozolol-17β-*O*-hemisuccinate (**1**) in only two steps, as shown in Scheme 1. The structural assignment of the products was made by ^1^H-NMR spectroscopy.

For the preparation of hemisuccinate **1**, we focused right away on the direct method using, as acylating agent, methyl 4-chloro-4-oxobutyrate that, as far as we know, had never been used before for this purpose (Scheme 1). In the preliminary experiments to introduce succinyl group in the reaction of **Stz** with methyl 4-chloro-4-oxobutyrate, we isolated a mixture of isomers, **2a** and **2b**, in an approximate 4:1 ratio, in accordance with the literature [36], that were not separated, as it was confirmed by ^1^H-NMR in Figure S1a,b (supplemental material). The ^1^H-NMR spectrum allowed us to distinguish between the two regioisomers. In particular, the shift of 5′ hydrogen of the pyrazole ring in the major isomer **2a**, acylated in *N*-1′ position, was 7.91 ppm, whereas, for the regioisomer **2b**, acylated in *N*-2′ position, the shift was lower, at 7.44 ppm. The deshifted 7.91 ppm in the case of isomer **2a** was caused by the anisotropic effect of the succinyl substitute in the *N*-1′ position. The shift is less evident in the case of the isomer **2b**, due to the distance of the succinate in *N*-2′ position compared to 5′-H. The identity of the two regioisomers was also confirmed because the presence of succinate in the pyrazole ring affected the hydrogens in 1-H and 4-H positions of the steroidic ring. In fact, if in the isomer **2a** the 1-H and 4-H shifts are substantially unchanged compared to the shifts of the analogous hydrogens in **Stz**, in the isomer **2b** the 4β-H shift is deshifted from 2.61 to 3.08 ppm and the 4α-H from 2.28 to 2.48 ppm, due to the closeness of succinate. All spectra and the comparative spectra of mixture **2a/2b** are reported in the Supplemental Material Figure S1a and 1bS.

Without intermediates **2a** and **2b** isolation and purification, the two successive additions of the same quantities (1.33 eq each) of methyl 4-chloro-4-oxobutyrate after 24 and 48 h gave the compounds **3a** and **3b**. In spite of the inert character of the tertiary alcohol in the 17β position, we were able to obtain the ester in this hindered position. The ^1^H-NMR spectrum of the mixture confirmed the introduction of a second succinate moiety (Supplemental Material).

Subsequent treatment of **3a** and **3b** mixture with NaOH in methanol at room temperature gave first the methyl steroid succinate (**4**), not isolated, and then the derivative **1** [19]. In compound **4,** the removal of the succinate from the two regioisomers was confirmed with the NMR spectrum, where it was observed that the shift of 5′-H, 1-H, and 4-H were at the same position of **Stz**.

Compounds **2a** and **2b** and **4** have only been isolated and purified once for the deep NMR characterization to study the intermediates of the reaction that lead to the final product. Thus, by means of this sequence of only two reaction steps, we were able to obtain excellent overall yields (55%) of desired **Stz** hemisuccinate (**1**).

## 3. Material and Methods

### 3.1. Chemistry

Chemicals, solvents, and standards were obtained from Sigma–Aldrich (Merck, Darmstadt, Germany) and used without further purification. Stanozolol was purchased from Steraloids (Steraloids Inc., Newport, RI, USA). Column chromatography was performed on Silica Gel 60 (70–230 mesh) using the specified eluents. The progress of the reactions was monitored by analytical thin-layer chromatography (TLC) on pre-coated glass plates (silica gel 60 F254-plate-Merck, Darmstadt, Germany) and the products were visualized by UV light. The purity of all compounds (>99%) was verified by thin-layer chromatography and NMR measurements [37,38,39,40].

### 3.2. NMR Analysis

NMR spectra were registered on a Bruker AVANCE 500 spectrometer (Bruker Italia Srl, Milan, Italy) equipped with a 5-mm broadband reverse probe and deuterium lock with field z-gradient operating at 500.13 and 125.76 MHz for ^1^H and ^13^C, respectively. All NMR spectra were recorded at 298 K in CDCl_3_ (isotopic enrichment 99.98%) solution, and the chemical shifts were reported on a *δ* (ppm) scale. The central peak of DMSO-*d*_6_ signals (2.49 ppm for ^1^H and 39.50 ppm for ^13^C) and of CDCl_3_ signals (7.28 ppm for ^1^H and 77.7 ppm for ^13^C) were used as the internal reference standard. Acquisition parameters for 1D were as follows: ^1^H spectral width of 5000 Hz and 32 K data points providing a digital resolution of ca. 0.305 Hz per point, relaxation delay 2 s; ^13^C spectral width of 29,412 Hz and 64 K data points providing a digital resolution of ca. 0.898 Hz per point, relaxation delay 2.5 s. The experimental error in the measured ^1^H-^1^H coupling constants was ± 0.5 Hz.

### 3.3. Synthesis of Compound 3 

To a suspension of **Stz** (200 mg, 0.6 mmol) and dimethylaminopyridine in anhydrous dichloromethane (5 mL), methyl-4-chloro-4-oxo-butyrate (100 μL, 0.8 mmol) was slowly added. Two successive portions of the same quantity of methyl-4-chloro-4-oxo-butyrate were added after 24 h and 48 h, and stirring was continued until the completion of the reaction (overall 72 h). The reaction was monitored by TLC (dichloromethane/ methanol 95:5). The mixture was poured in water and extracted with dichloromethane (3 × 5 mL), and the extracts were washed with HCl 0.1 M. The solution was then concentrated under vacuum, and hexane was added. The precipitate was separated by filtration on celite, and the residue was evaporated, giving 300 mg of an uncolored oil (0.54 mmol, yield 90%).

To verify the identity of intermediates **2a** and **2b** by NMR, the reaction was stopped after 2 h, and the mixture was separated on column chromatography on silica gel (petroleum ether/ethyl acetate 7:3).

Mixture **2a** and **2b**: R_f_ 0.7 (dichloromethane/methanol 95:5); ^1^H NMR (CDCl_3_) major regioisomer **2a**: δ 7.91 (1H, s, 5′-H), 3.72 (3H, s, COOC*H*_3_), 3.41 (2H, t, *J* = 6.5 Hz, NCOC*H*_2_CH_2_COOCH_3_), 2.78 (2H, t, J = 6.5 Hz, NCOCH_2_C*H*_2_COOCH_3_), 2.71 (1H, d, *J* = 15.4 Hz, 1β-H), 2.68 (1H, dd, *J* = 12.2, 2.0 Hz, 4β-H), 2.33 (1H, dd, *J* = 12.2, 2.0 Hz, 4α-H), 2.15 (1H, d, *J* = 15.4 Hz, 1α-H), 1.28 (3H, s, 20-C*H*_3_), 0.89 (3H, s, 19-C*H*_3_), 0.79 (3H, s, 18-C*H*_3_); minor regioisomer **2b**: δ 7.44 (1H, s, 5′-H), 3.73 (3H, s, COOC*H*_3_), 3.46 (2H, t, *J* = 6.5 Hz, NCOC*H*_2_CH-COOCH_3_), 3.08 (1H, dd, *J* = 12.2, 2.0 Hz, 4β-H), 2.76 (2H, t, *J* = 6.5 Hz, NCOCH_2_C*H*_2_COOCH_3_), 2.53 (1H, d, J = 15.4 Hz, 1β-H), 2.48 (1H, dd, J = 12.2, 2.0 Hz, 4α-H), 2.07 (1H, d, *J* = 15.4 Hz, 1α-H), 1.25 (3H, s, 20-C*H*_3_), 0.89 (3H, s, 19-C*H*_3_), 0.76 (3H, s, 18-C*H*_3_)

Mixture **3a** and **3b**: R_f_ 0.9 (dichloromethane/methanol 95:5); ^1^H NMR (CDCl_3_) major regioisomer **3a**: δ 7.91 (1H, s, 5′-H), 3.72 and 3.70 (6H, s, 2 × COOC*H*_3_), 3.41 (2H, t, *J* = 6.5 Hz, NCOC*H*_2_CH_2_-COOCH_3_), 2.78 (2H, t, *J* = 6.5 Hz, NCOCH_2_C*H*_2_COOCH_3_), 2.71 (1H, d, *J* = 15.4 Hz, 1β-H), 2.68 (1H, dd, *J* = 12.2, 2.0 Hz, 4β-H), 2.66–2.52 (4H, m, NCOC*H*_2_C*H*_2_COO), 2.33 (1H, dd, *J* = 12.2, 2.0 Hz, 4α-H), 2.07 (1H, d, *J* = 15.4 Hz, 1α-H), 1.27 (3H, s, 20-C*H*_3_), 0.87 (3H, s, 19-C*H*_3_), 0.77 (3H, s, 18-C*H*_3_); minor regioisomer **3b**: δ 7.44 (1H, s, 5′-H), 3.73 and 3.70 (6H, s, 2 × COOC*H*_3_), 3.45 (2H, t, *J* = 6.5 Hz, NCOC*H*_2_CH_2_-COOCH_3_), 2.76 (2H, t, *J* = 6.5 Hz, NCOCH_2_C*H*_2_COOCH_3_), 2.66–2.52 (5H, m, NCOC*H*_2_C*H*_2_COO and 1β-H), 2.48 (1H, dd, J=12.2, 2.0 Hz, 4β-H), 2.33 (1H, dd, *J* = 12.2, 2.0 Hz, 4α-H), 2.07 (1H, d, *J* = 15.4 Hz, 1α-H), 1.27 (3H, s, 20-C*H*_3_), 0.87 (3H, s, 19-C*H*_3_), 0.75 (3H, s, 18-C*H*_3_).

### 3.4. Synthesis of 17β-O-hemisuccinate of Stanozolol (1)

To a solution of **3a**/**3b** in methanol (3 mL) powdered NaOH was added in three portions of 20 mg each during 48 h. Stirring at room temperature was continued for other 24 h (overall 72 h). After TLC (dichloromethane/methanol 95:5) the resulting precipitate was collected under vacuum as a white solid (140 mg). The solid was resuspended in methanol and acidic resin (Dowex 50W × 8) was added (20 mg). After 24 h at room temperature under stirring, filtration and evaporation of the solvent gave 140 mg of product as white solid (0.3 mmol, overall yield 55%).

Only to perform NMR analysis, compound **4** was isolated by column chromatography on silica gel (dichloromethane/methanol 95:5), after 1 h.

Compound **4**: R_f_ 0.5 (dichloromethane/methanol 95:5); ^1^H NMR (CDCl_3_) δ 7.32 (1H, s, 5′-H), 3.71 (3H, s, COOC*H*_3_), 2.64 (1H, d, *J* = 15.4 Hz, 1β-H), 2.66–2.57 (5H, m, NCOC*H*_2_C*H*_2_COO and 4β-H), 2.30 (1H, dd, J = 12.2, 2.0 Hz, 4α-H), 2.11 (1H, d, *J* = 15.4 Hz, 1α-H), 1.28 (3H, s, 20-C*H*_3_), 0.88 (3H, s, 19-C*H*_3_), 0.78 (3H, s, 18-C*H*_3_).

Compound **1**: R_f_ 0.1 (dichloromethane/methanol 95:5), mp 218–220 °C (from MeOH) (lett. 215–217 °C [33]). ^1^H-NMR (DMSO-*d*_6_): δ 7.23 (1H, s, 5′-H), 3.58 (1H, s, NH); 2.55–2.40 (6H, m, OCOC*H*_2_C*H*_2_COOH, 1β-H and 4β-H), 2.14 (1H, dd, *J* = 12.2, 2.0 Hz, 4α-H), 2.05 (1H, d, *J* = 15.4 Hz, 1α-H), 1.35 (3H, s, 17-CH_3_), 0.82 (3H, s, 18-CH_3_), 0.68 (3H, s, 19-CH_3_). ^13^C-NMR (DMSO-*d*_6_): δ 173.9 (COOH), 171.5 (OCOCH_2_), 141.5 (C-3), 131.6 (C-5′), 113.6 (C-2), 90.7 (C-17), 53.4, 51.8, 48.8, 46.5, 42.5, 36.6, 36.3, 35.0, 32.2, 31.6, 30.2, 29.4 (-OCO*C*H_2_CH_2_COOH), 29.3 (-OCOCH_2_*C*H_2_COOH), 26.7, 23.8, 21.6 (CH_3_-17), 20.7 (C-11), 14.6 (C-19), 11.8 (C-18).

## 4. Conclusions

To summarize, we propose here an alternative procedure for the synthesis of 17β-*O*-hemisuccinate of stanozolol (**1**). The new approach involves a sequence of two simultaneous transformations without a preventive protection of the highly reactive pyrazole-nitrogen atoms. Indeed in the first step, stanozolol is transformed in the disuccinate **3a,b** then, in a one-pot reaction, the successive treatment with NaOH/MeOH gives the simultaneous deprotection of the pyrazole ring and the hydrolysis of the 17β-methyl ester. Our method gives good yields and has the advantage that tedious purification and isolation of the intermediates are not necessary.

## Supplementary Materials

The following are available online, Figure S1a. 1H-NMR spectrum from 8.40 ppm to 6.00 ppm of Stanozolol (top) and mixture 2a/2b (bottom). Figure S1b. 1H-NMR spectrum from 4.20 ppm to 2.00 ppm of Stanozolol (top) and mixture 2a/2b (bottom).

## References

[1] Kayser B., Mauron A., Miah A. (2007). Current anti-doping policy: A critical appraisal. BMC Med. Ethics.

[2] Lu H., Conneely G., Pravda M., Guilbault G.G. (2006). Screening of boldenone and methylboldenone in bovine urine using disposable electrochemical immunosensors. Steroids.

[3] Popii V., Baumann G. (2004). Laboratory measurement of growth hormone. Clin. Chim. Acta.

[4] Rúbies A., Cabrera A., Centrich F. (2007). Determination of synthetic hormones in animal urine by high-performance liquid chromatography/mass spectrometry. J. AOAC Int..

[5] Kolmonen M., Leinonen A., Pelander A., Ojanperä I. (2007). A general screening method for doping agents in human urine by solid phase extraction and liquid chromatography/time-of-flight mass spectrometry. Anal. Chim. Acta.

[6] Thevis M., Schänzer W. (2007). Mass spectrometry in sports drug testing: Structure characterization and analytical assays. Mass Spectrom. Rev..

[7] Rocha D.G., Lana M.A.G., Augusti R., Faria A.F. (2019). Simultaneous Identification and Quantitation of 38 Hormonally Growth Promoting Agent Residues in Bovine Muscle by a Highly Sensitive HPLC-MS/MS Method. Food Anal. Methods.

[8] Chiesa L.M., Nobile M., Panseri S., Biolatti B., Cannizzo F.T., Pavlovic R., Arioli F. (2017). Bovine teeth as a novel matrix for the control of the food chain: Liquid chromatography-tandem mass spectrometry detection of treatments with prednisolone, dexamethasone, estradiol, nandrolone and seven β2-agonists. Food Addit. Contam. Part A Chem. Anal. Control Expo. Risk Assess..

[9] Li C., Wang Z., Cao X., Beier R.C., Zhang S., Ding S., Li X., Shen J. (2008). Development of an immunoaffinity column method using broad-specificity monoclonal antibodies for simultaneous extraction and cleanup of quinolone and sulfonamide antibiotics in animal muscle tissues. J. Chromatogr. A.

[10] Zhao S., Li X., Ra Y., Li C., Jiang H., Li J., Qu Z., Zhang S., He F., Wan Y. (2009). Developing and optimizing an immunoaffinity cleanup technique for determination of quinolones from chicken muscle. J. Agric. Food Chem..

[11] Wang J., Zheng L., Dong Y., Song Z., Wang Y., Meng M., Ren L., Eremin S.A., Deng C., Yin Y. (2016). Establishment of Enhanced Chemiluminescent Immunoassay Formats for Stanozolol Detection in animal-derived foodstuffs and Other Matrices. Food Anal. Methods.

[12] Erlanger B.F. (1980). The preparation of antigenic Hapten-Carrier conjugates: A survey. Immunochemical Techniques, Part A.

[13] Butt W.R. (1984). Practical Immunoassay: The State of the Art.

[14] Drašar P., Černý I., Pouzar V., Havel M. (1984). New preparation of steroidal 3-hemisuccinates. Collect. Czech. Chem. Commun..

[15] Zingg M., Meyer K. (1957). Succinic acid monoesters of some digitaloid aglycones. Pharm. Acta Helv..

[16] Chen H.-X., Zhang X.-X. (2008). Antibody development to testosterone and its application in capillary electrophoresis-based immunoassay. Electrophoresis.

[17] Orita A., Tanahashi C., Kakuda A., Otera J. (2001). Highly powerful and practical acylation of alcohols with acid anhydride catalyzed by Bi(OTf)(3). J. Org. Chem..

[18] Ottolina G., Carrea G., Riva S. (1991). Regioselective Enzymatic Preparation of Hemisuccinates of Polyhydroxylated Steroids. Biocatalysis.

[19] Evans D.D., Evans D.E., Lewis G.S., Palmer P.J., Weyell D.J. (1963). 674. A novel method for the preparation of steroid esters. J. Chem. Soc..

[20] Clinton R.O., Manson A.J., Stonner F.W., Beyler A.L., Potts G.O., Arnold A. (1959). STEROIDAL [3,2-c]PYRAZOLES. J. Am. Chem. Soc..

[21] Poelmans S., De Wasch K., De Brabander H., Van De Wiele M., Courtheyn D., Van Ginkel L., Sterk S., Delahaut P., Dubois M., Schilt R. (2002). Analytical possibilities for the detection of stanozolol and its metabolites. Anal. Chim. Acta.

[22] Yesalis C.E., Bahrke M.S. (1995). Anabolic-androgenic steroids. Current issues. Sports Med..

[23] Rubegni P., Flori M.L., Fimiani M., Andreassi L. (1996). A case of cryofibrinogenaemia responsive to stanozolol. Br. J. Haematol..

[24] Berger J.R., Pall L., Winfield D. (1993). Effect of anabolic steroids on HIV-related wasting myopathy. South. Med. J..

[25] Sheffer A., Fearon D., Frankausten K. (1987). Hereditary angioedema: A decade of management with stanozolol. J. Allergy Clin. Immunol..

[26] Graham M.R., Ryan P., Baker J.S., Davies B., Thomas N.-E., Cooper S.-M., Evans P., Easmon S., Walker C.J., Cowan D. (2009). Counterfeiting in performance- and image-enhancing drugs. Drug Test. Anal..

[27] Thevis M., Kuuranne T., Geyer H. (2018). Annual banned-substance review: Analytical approaches in human sports drug testing. Drug Test. Anal..

[28] Liu Y., Lu J., Yang S., Xu Y., Wang X. (2015). A new potential biomarker for 1-testosterone misuse in human urine by liquid chromatography quadruple time-of-flight mass spectrometry. Anal. Methods.

[29] Huml L., Havlová D., Longin O., Staňková E., Holubová B., Kuchař M., Prokudina E., Rottnerová Z., Zimmermann T., Drašar P. (2020). Stanazolol derived ELISA as a sensitive forensic tool for the detection of multiple 17α-methylated anabolics. Steroids.

[30] Salvador J.-P., Sánchez-Baeza F., Marco M.-P. (2010). A high-throughput screening (HTS) immunochemical method for the analysis of stanozolol metabolites in cattle urine samples. J. Chromatogr. B Analyt. Technol. Biomed. Life Sci..

[31] Tsitsimpikou C., Tsarouhas K., Spandidos D.A., Tsatsakis A.M. (2016). Detection of stanozolol in the urine of athletes at a pg level: The possibility of passive exposure. Biomed. Rep..

[32] Thevis M., Fußhöller G., Geyer H., Rodchenkov G., Mareck U., Sigmund G., Koch A., Thomas A., Schänzer W. (2006). Detection of Stanozolol and Its Major Metabolites in Human Urine by Liquid Chromatography-Tandem Mass Spectrometry. Chroma.

[33] Evans D.D., Palmer P.J. (1965). Steroid esters of dibasic acids. Steroids.

[34] Longin O., Černý I., Drašar P. (2015). Novel approach to the preparation of hemisuccinates of steroids bearing tertiary alcohol group. Steroids.

[35] Clinton R.O., Manson A.J., Stonner F.W., Neumann H.C., Christiansen R.G., Clarke R.L., Ackerman J.H., Page D.F., Dean J.W., Dickinson W.B. (1961). Steroidal[3,2-c]pyrazoles. II. 1 Androstanes, 19-Norandrostanes and their Unsaturated Analogs. J. Am. Chem. Soc..

[36] Christiansen R.G., Bell M.R., D’Ambra T.E., Mallamo J.P., Herrmann J.L., Ackerman J.H., Opalka C.J., Kullnig R.K., Winneker R.C., Snyder B.W. (1990). Antiandrogenic steroidal sulfonylpyrazoles. J. Med. Chem..

[37] Ciuffreda P., Casati S., de Mieri M., Ferraboschi P. (2009). Corticosteroids 21-glucuronides: Synthesis and complete characterization by 1H and 13C NMR. Steroids.

[38] Ciuffreda P., Casati S., Manzocchi A. (2004). Complete 1H and 13C NMR spectral assignment of 17-hydroxy epimeric sterols with planar A or A and B rings. Magn. Reson. Chem..

[39] Casati S., Ottria R., Ciuffreda P. (2009). 17alpha- and 17beta-boldenone 17-glucuronides: Synthesis and complete characterization by 1H and 13C NMR. Steroids.

[40] Ottria R., Casati S., Ciuffreda P. (2012). 1H, 13C and 15N NMR assignments for N- and O-acylethanolamines, important family of naturally occurring bioactive lipid mediators. Magn. Reson. Chem..

