# Supplementary material for: Simple Synthesis of 17-β-O-hemisuccinate of Stanozolol for Immunoanalytical Methods
Source: Molecules. 2020 Apr 26;25(9):2019. doi: 10.3390/molecules25092019 (PMC7248714; doi:10.3390/molecules25092019)
Supplement: Supplementary file 1 [file molecules-25-02019-s001.pdf]

Supplemental Material

Simple synthesis of 17-β-O-hemisuccinate of stanozolol for immunoanalytical methods

Silvana Casati[a], Roberta Ottria[a], Pierangela Ciuffreda[a #]

[a] *Dipartimento di Scienze Biomediche e Cliniche "Luigi Sacco", Università degli Studi di Milano, Via G.B. Grassi 74, 20157 Milano, Italy*

silvana.casati@unimi.it, roberta.ottria@unimi.it; pierangela.ciuffreda@unimi.it

Table of Contents

Figures

Figure S1a/S1b Pag. 2

^{1}H and ^{13}C-NMR data

^{1}H spectrum of mixture **2a/2b** Pag. 3

^{1}H spectrum of mixture **3a/3b** Pag. 4

^{1}H spectrum of mixture **4** Pag. 5

^{1}H and ^{13}C NMR spectra of stanazolol 17β-*O*-hemisuccinate (**1**) Pag. 6

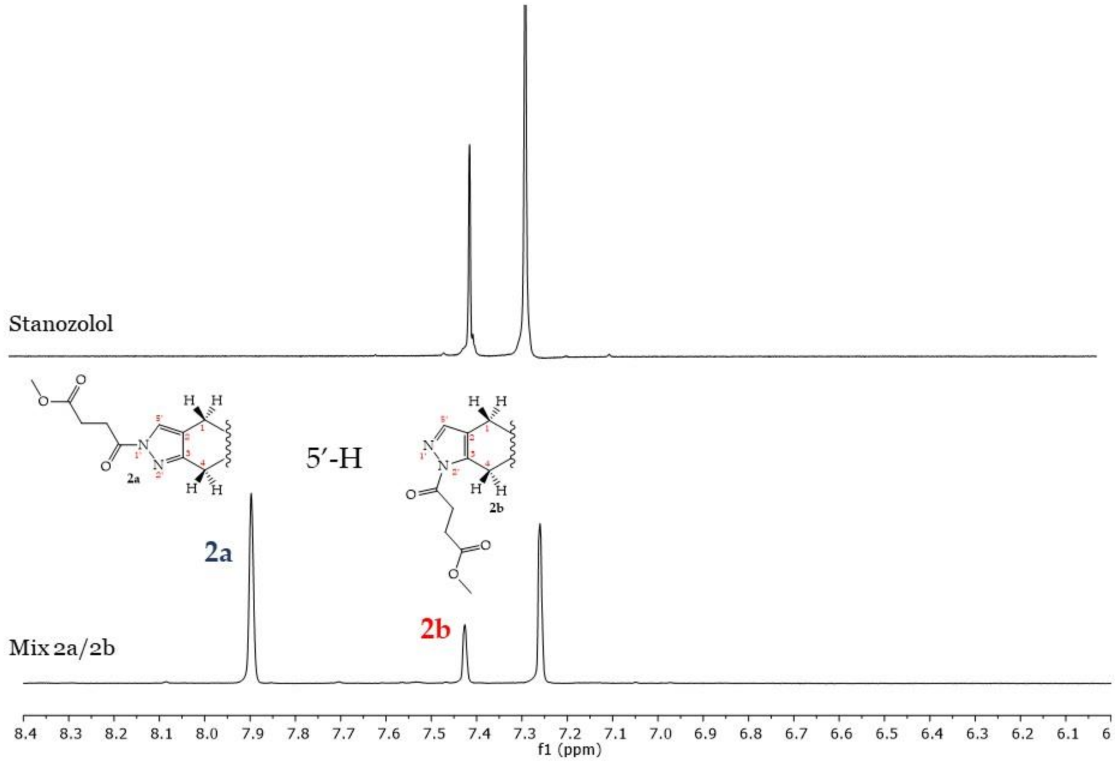

Figure S1a. ^{1}H-NMR spectrum from 8.40 ppm to 6.00 ppm of Stanozolol (top) and mixture **2a/2b** (bottom).

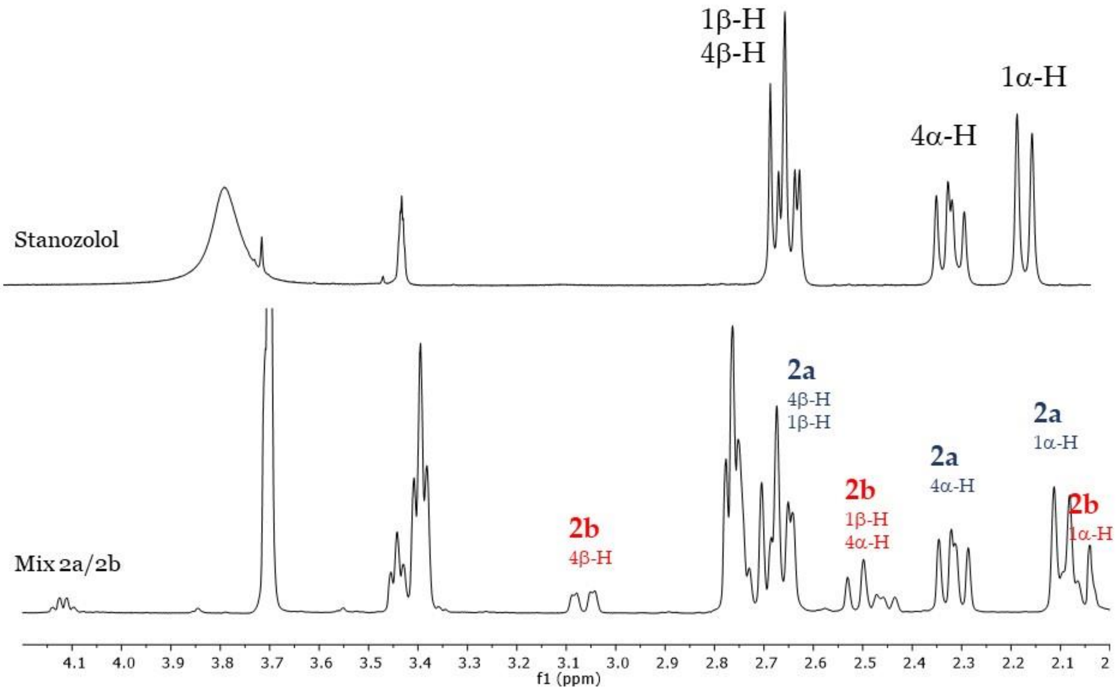

Figure S1b. ^{1}H-NMR spectrum from 4.20 ppm to 2.00 ppm of Stanozolol (top) and mixture **2a/2b** (bottom).

^{1}H spectrum of mixture **2a**/**2b**

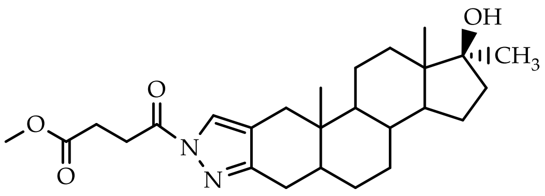

2a

4:1

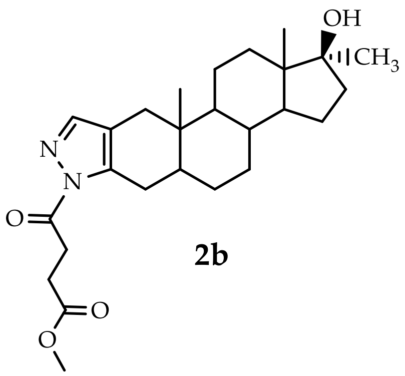

2b

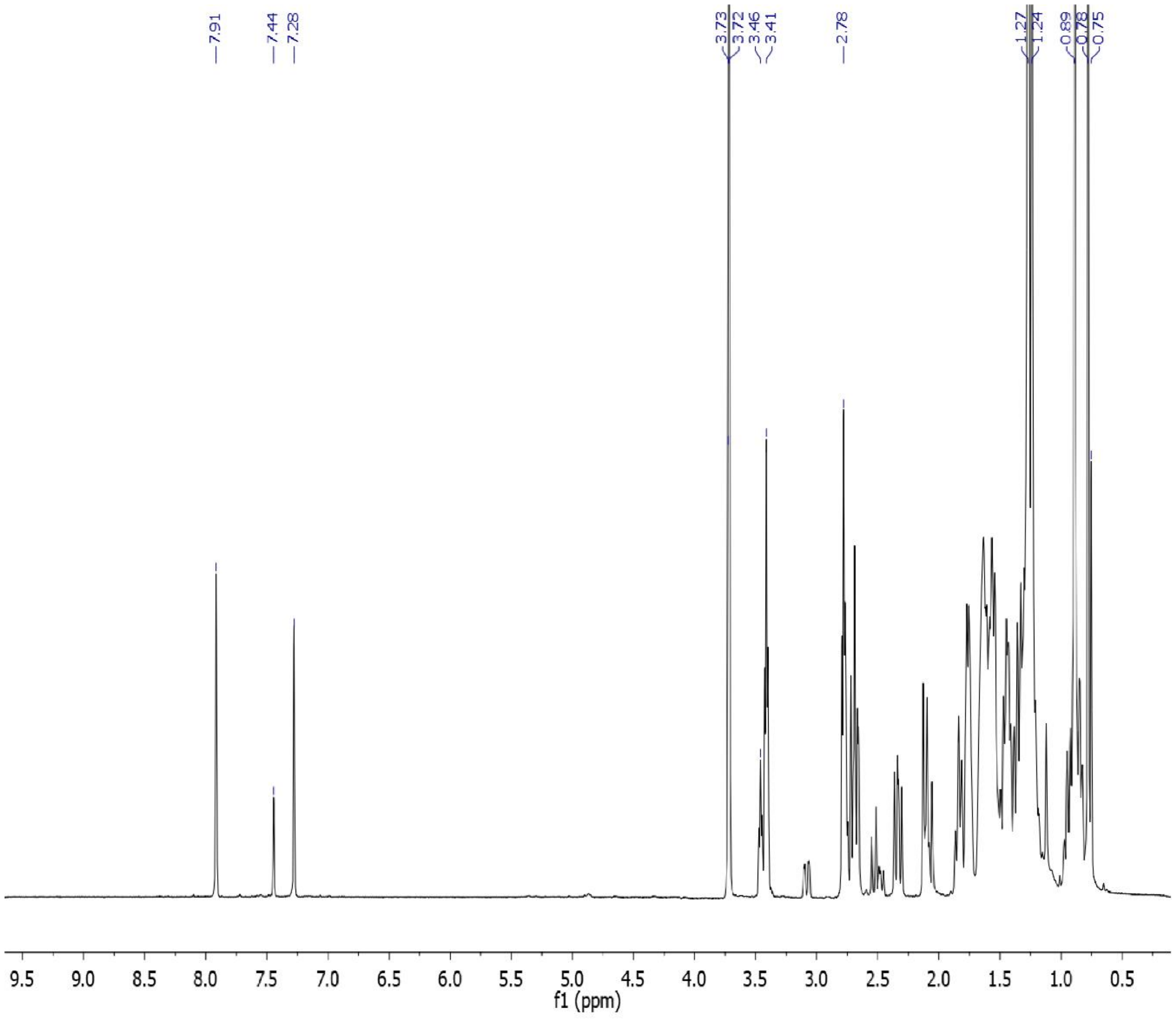

^{1}H spectrum of mixture **3a**/**3b**

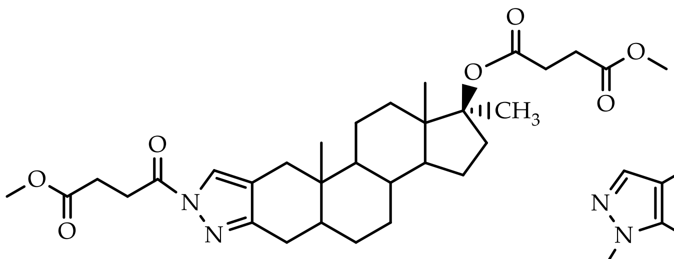

3a

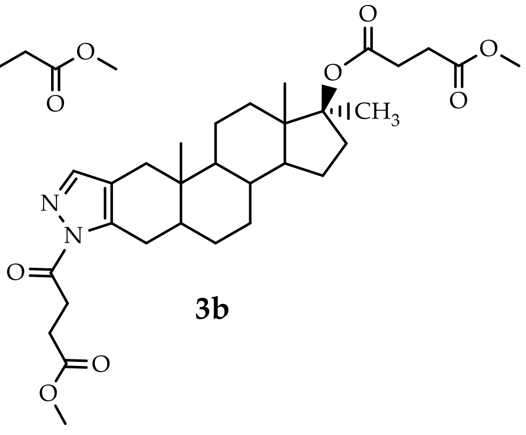

3b

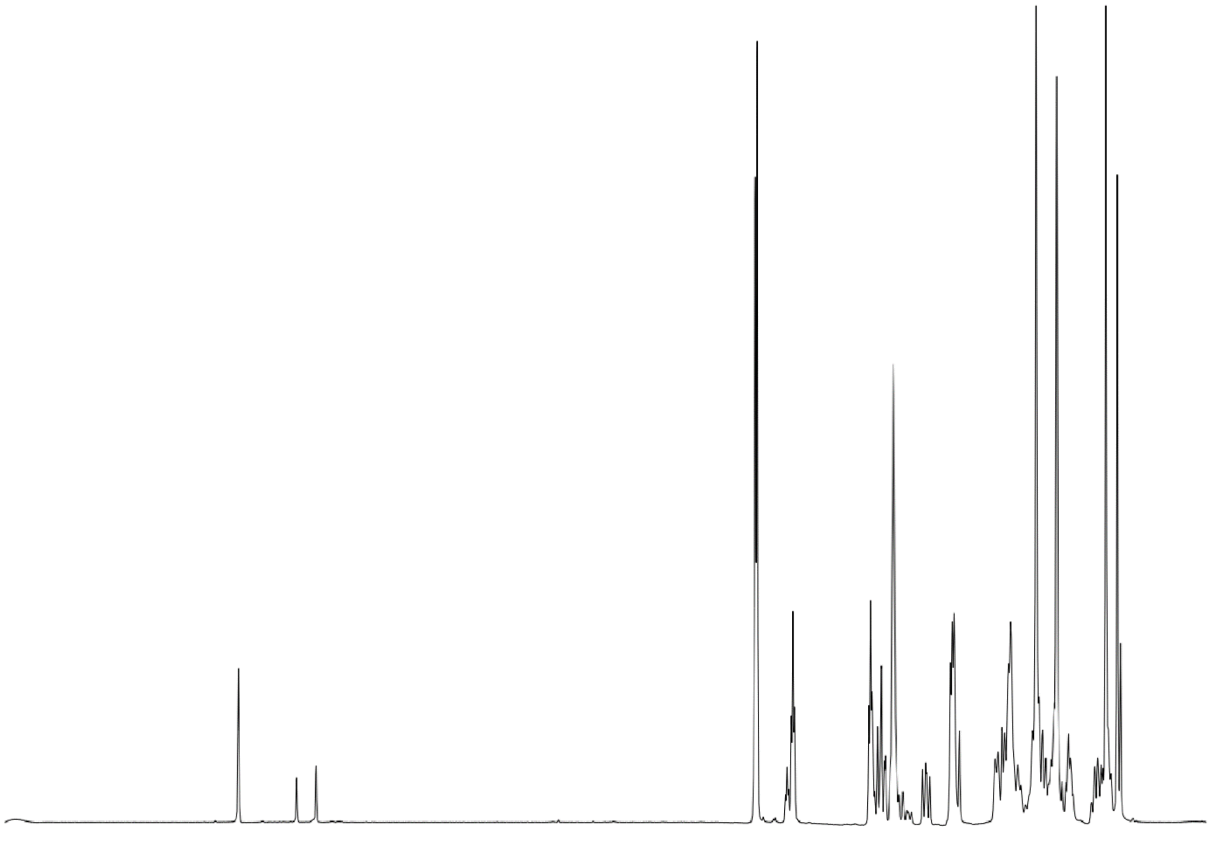

^{1}H spectrum of compound **4**

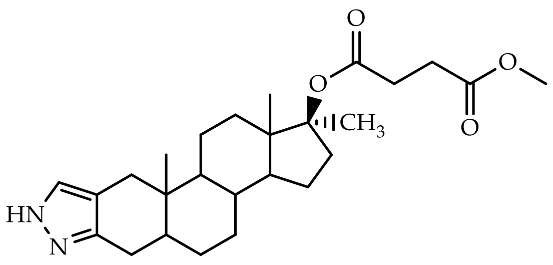

4

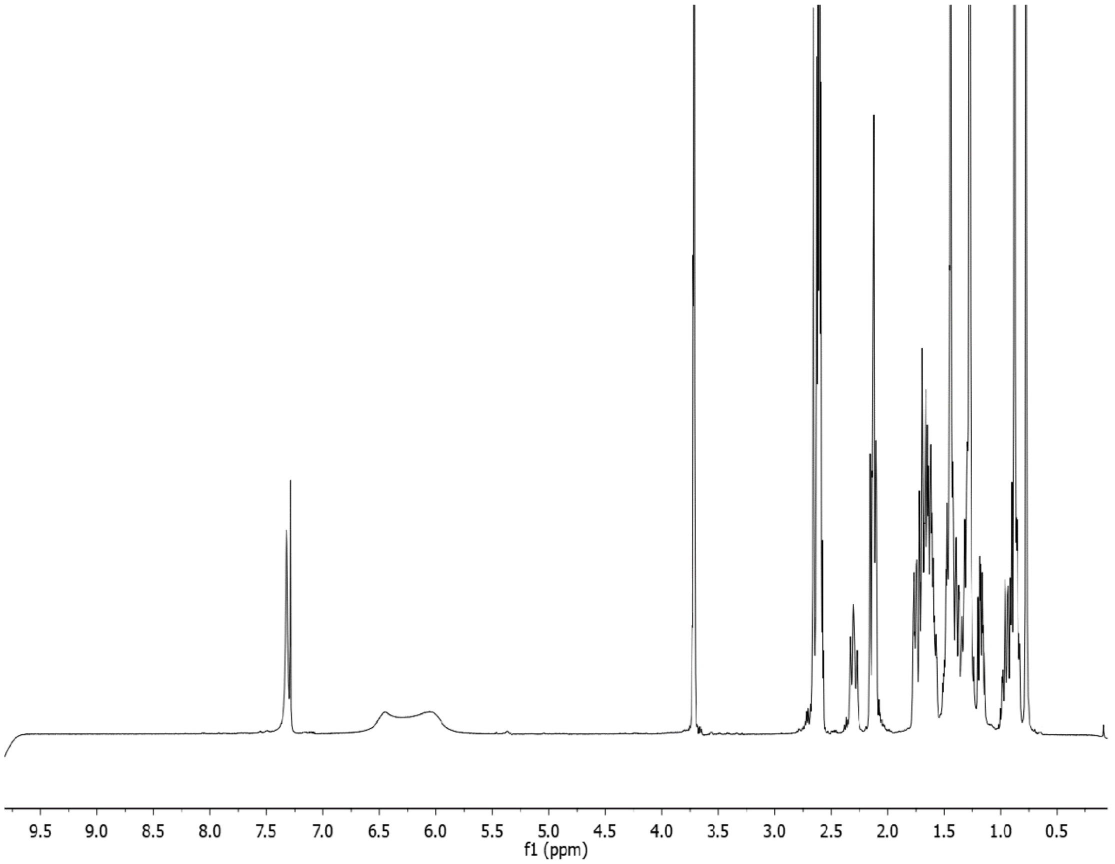

^{1}H and ^{13}C NMR spectra of stanazolol 17β-*O*-Hemisuccinate (**1**)

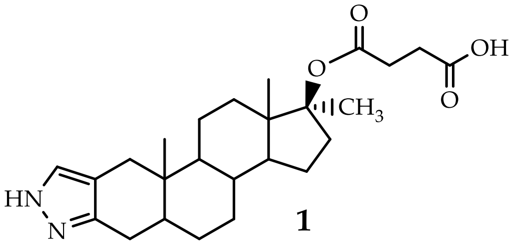

1

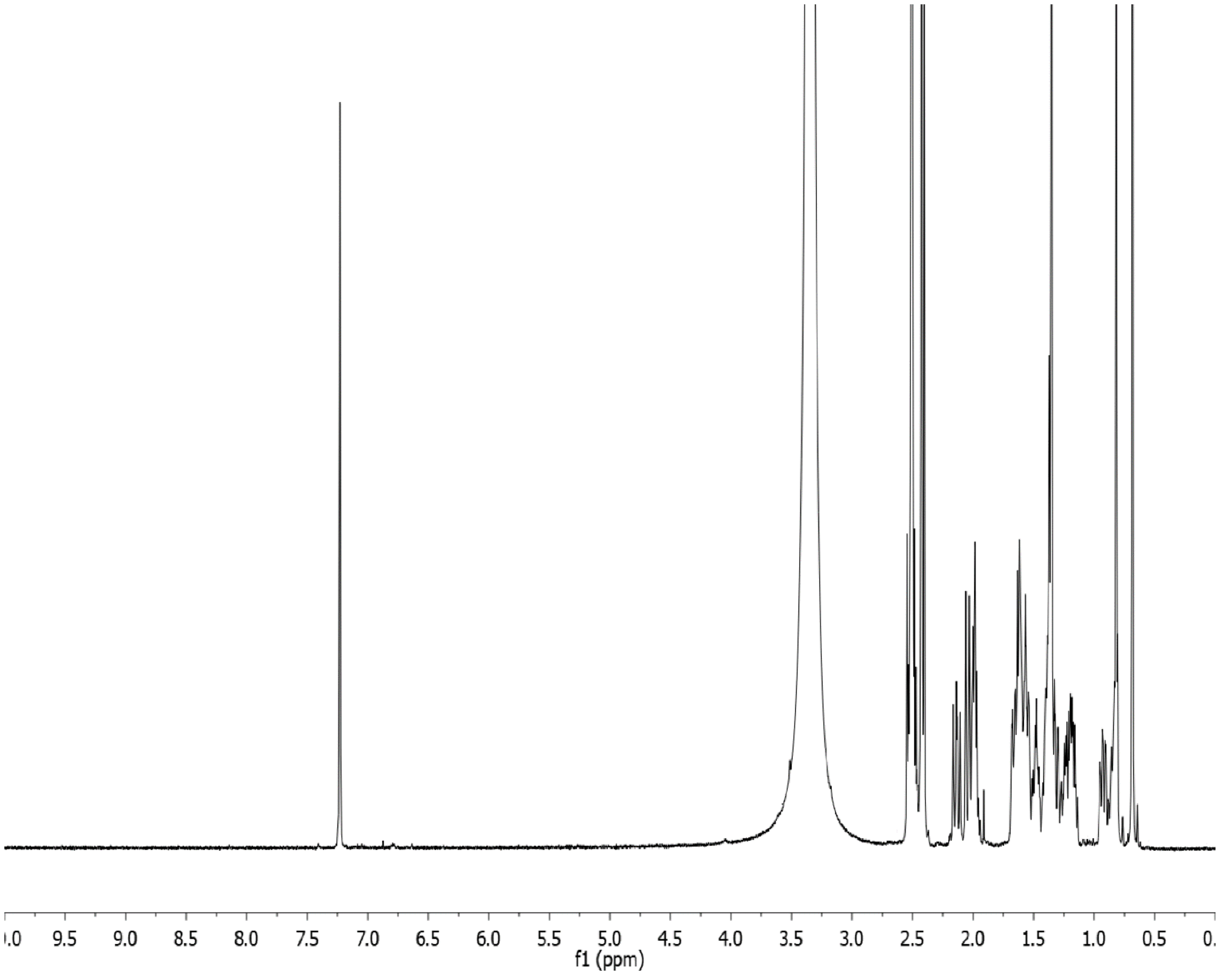

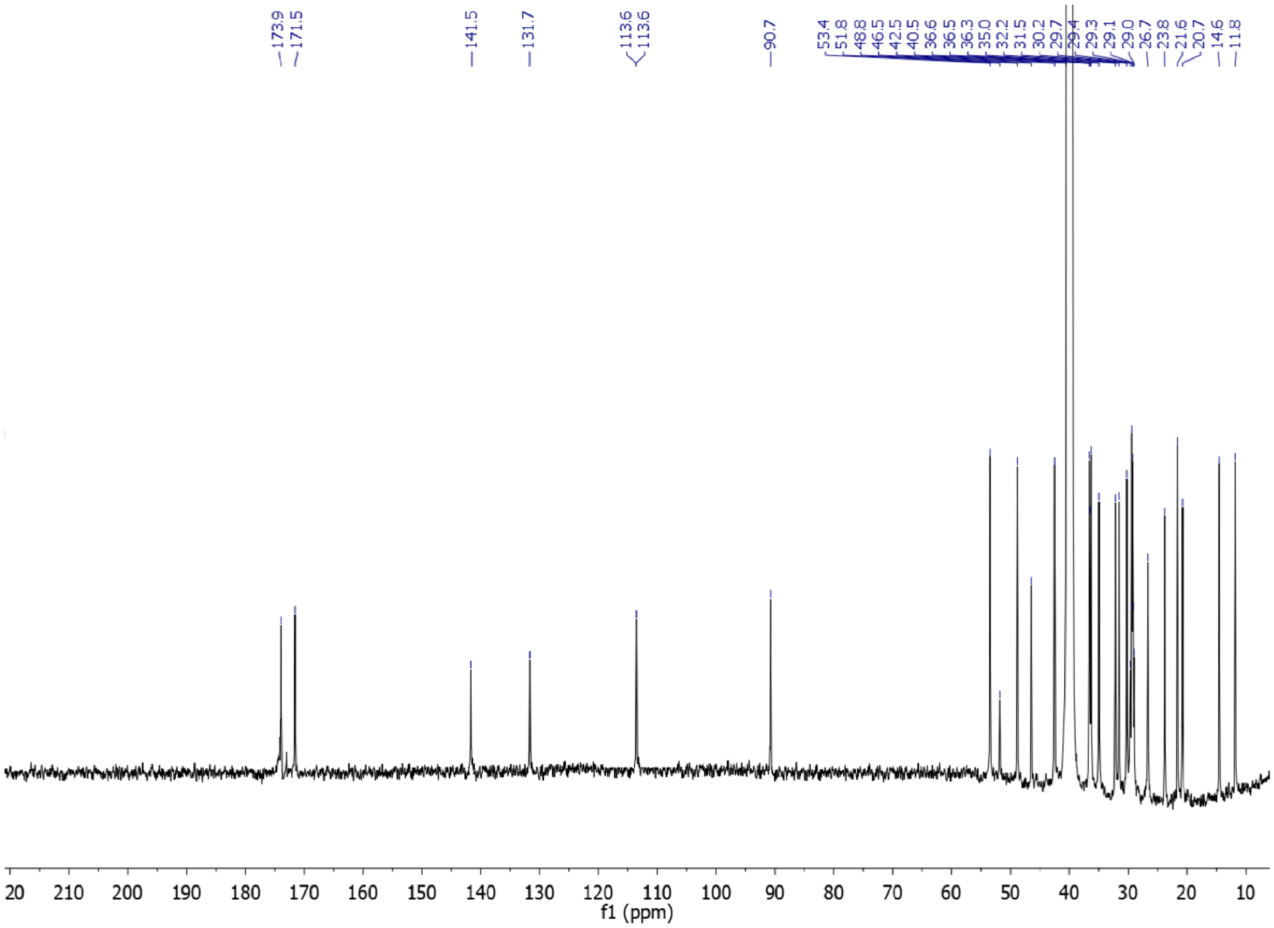
173.9
171.5
141.5
131.7
113.6
113.6
90.7
53.4
51.8
48.8
46.5
42.5
40.5
36.6
36.5
36.3
35.0
32.2
31.5
30.2
29.7
29.4
29.3
29.1
29.0
26.7
23.8
21.6
20.7
14.6
11.8
20 210 200 190 180 170 160 150 140 130 120 110 100 90 80 70 60 50 40 30 20 10
f1 (ppm)
